# Reversible dilatation of lumbar epidural venous plexus secondary to pelvic venous compression: mimicker of pathology

**DOI:** 10.1259/bjrcr.20150287

**Published:** 2016-08-25

**Authors:** August S Tuan, S Ali Nabavizadeh, Bryan Pukenas, Suyash Mohan, Kim O Learned

**Affiliations:** Department of Radiology, Hospital of the University of Pennsylvania, Philadelphia, PA, USA

## Abstract

Radiculopathy from dilated epidural varices is a rarely recognized phenomenon, and engorged varices may mimic a disc herniation, fistula or mass. We report a case of a distended bladder causing reversible pelvic venous congestion and resultant epidural venous plexus engorgement. We show marked engorgement of the epidural venous plexus and crowding of the cauda equina from a markedly distended bladder with resolution of these findings following bladder decompression. Familiarity with this clinical entity is crucial to avoid misdiagnosis and to prevent potential unnecessary surgical intervention.

## Background

Radiculopathy from dilated epidural varices is rarely recognized, and often the varices mimic a mass or disc herniation.^1^ Epidural venous dilation from pregnancy,^2^ lymphadenopathy,^3^ absence or thrombosis of the inferior vena cava,^4^ intracranial hypotension,^5^ overshunting of cerebrospinal fluid (CSF)^6^ and hyperventilation^7^ can produce similar symptoms and have been previously described. There are prior case reports of this clinically unsuspected entity having been diagnosed during surgical exploration of these pseudo-discs.^1,4^

We report a case of reversible pelvic venous compression leading to epidural venous plexus engorgement from a distended urinary bladder, masquerading as a dural arteriovenous fistula (d-AVF) and/or pseudo-disc.

## Clinical presentation

A 37-year-old male with a history of poorly controlled Type II diabetes presented with an event concerning for seizure and exacerbation of bilateral lower extremity weakness. He also had rhabdomyolysis and acute kidney injury. He had suffered epileptic events in the past that had been attributed to hypoglycaemia and thus was not on any anti-epileptic medication. On the day of his presentation, he could not move his lower extremities but was not confused or sluggish. He did not have bowel/bladder incontinence or saddle anaesthesia. On physical examination, he had 0/5 lower extremity motor strength, decreased sensation distally with a T5 sensory level and areflexia (0/5 in the lower extremities and 1+ in the upper extremities). MRI of the spine was obtained in view of the symptoms present.

## Imaging findings

**Figure 1.**
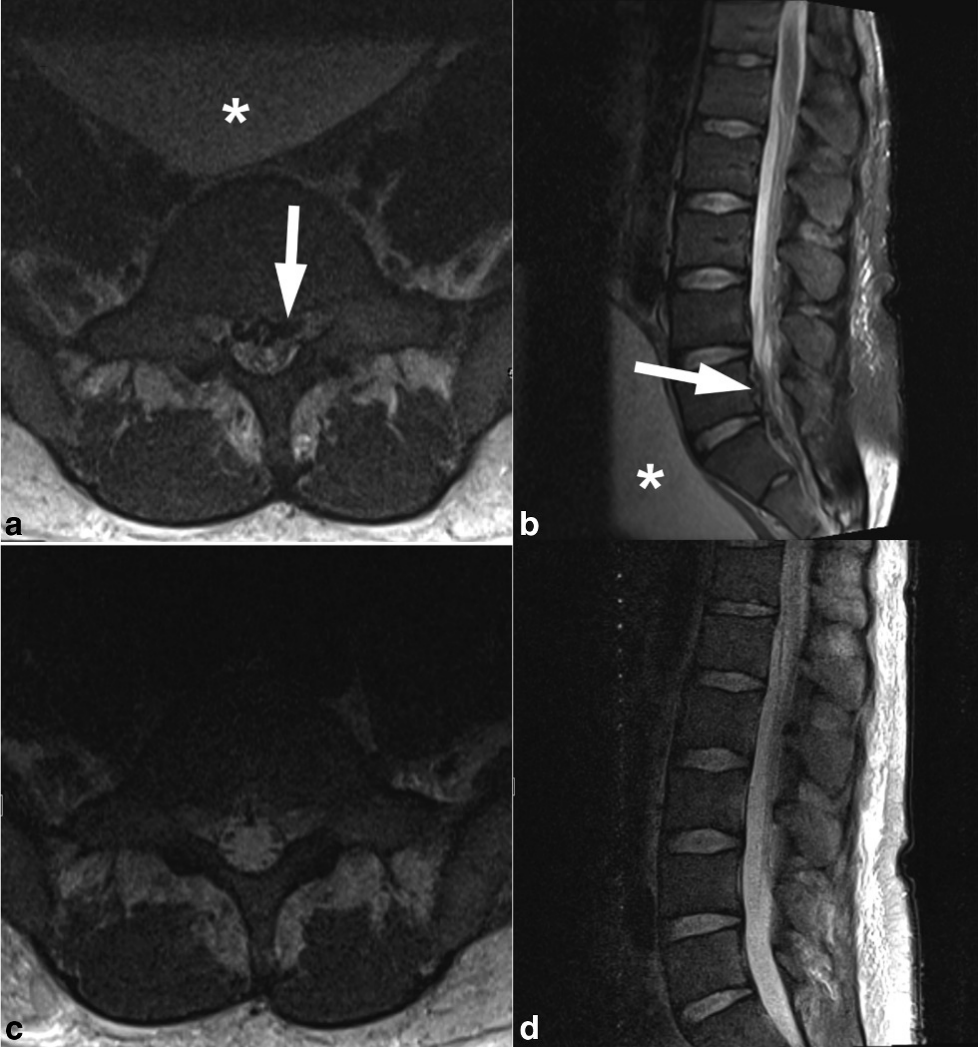
Axial and sagittal *T*_2_ weighted images (**a**, **b**) show marked engorgement of the epidural venous plexus (arrow), effacement of the cerebrospinal fluid space and crowding of the cauda equina. A distended urinary bladder is seen up against the lumbosacral spine (asterisk). Following bladder catheterization, axial and sagittal *T*_2_ weighted images (**c**, **d**) demonstrate resolution of epidural venous plexus engorgement resulting in a patent spinal canal.

Axial and sagittal *T*_2_ weighted images showed marked engorgement of the epidural venous plexus with effacement of the CSF space causing crowding of the cauda equina at the level of L5–S1 (Figure 1a, b). A markedly distended bladder was also present (Figure 1b). A urinary catheter was placed, which drained a large volume of urine.

A follow-up complete spine MRI and MR angiography were performed the next day. Axial and sagittal *T*_2_ weighted images showed a decompressed urinary bladder and resolution of the engorged epidural venous plexus, with improved patency of the spinal canal (Figure 1c, d). The spinal cord showed extensive, predominantly central, non-enhancing *T*_2_ hyperintensity extending from C2 to T11–12. No d-AVF was identified on the dynamic contrast-enhanced MR angiography.

## Further work-up and outcome

A lumbar puncture was performed, which was notable for CSF protein level over 600 mg dl^−1^ and a glucose level of 142 mg dl^−1^. Blood glucose level was 269 mg dl^−1^ and correlated with elevated CSF glucose. Extensive infectious and autoimmune work-up was unrevealing. Ultimately, a differential of neuromyelitis optica-like disease versus neurosarcoid versus other inflammatory disease was favoured. The patient subsequently received six total plasma exchange treatments with 5% albumin every other day, resulting in a mild improvement in his neurological symptoms.

Unfortunately, the patient became lethargic and then apnoeic during his hospitalization. Resuscitative efforts were performed but proved futile.

## Pathological findings

An autopsy revealed oedema and necrosis of the superior two-thirds of the spinal cord. The cauda equina roots were thickened. There was no epidural or subdural mass. The spinal meninges were thin and translucent and the subarachnoid space was free from exudate. The spinal arteries and veins were unremarkable.

## Discussion

Originally described by the French anatomist Gilbert Breschet, the vertebral venous system is a large, valveless and relatively low-pressure system running parallel to the vena cava, extending through the spinal column, and arranged in a longitudinal pattern as three distinct interconnecting divisions.^8^ Batson redefined the vertebral venous plexus into caval, pulmonary, portal and vertebral divisions and postulated that this system was a route for prostate cancer metastases.^8^ The lack of valves allows free communication between the internal and external venous networks.^9^ The vertebral venous plexus is considered a component of the cerebrospinal venous system, and its bidirectional flow allows regulation of intracranial pressure from postural and venous outflow changes.^8,9^

Normal venous return from the pelvis is through the vesical veins, which drain into the internal iliac veins before draining into the common iliac veins and into the inferior vena cava. If there is a large pelvic mass or pregnancy compressing the vessels, venous pressure may be elevated sufficiently to direct venous flow through collateral venous vessels owing to the bidirectional, valveless system.^2,7^ Venous return may then enter the vertebral venous plexus through intervertebral foraminal veins and engorge the anterior epidural space.^10^ Redirection of venous flow into the epidural veins causes engorgement of the vessels, distorting the walls of the dural sac and subsequently reducing the volume of the lumbosacral subarachnoid CSF, and crowding and compression of the cauda equina.^7^

In our patient, the markedly distended urinary bladder, secondary to transverse myelitis, compressed the pelvic venous system, resulting in engorgement of the lumbar spine epidural venous plexus through the valveless foraminal veins. Following catheterization of the bladder, the mass effect of the bladder on the pelvic veins resolved. This allowed normal venous return through the pelvic veins and subsequent resolution of epidural venous plexus engorgement.

This report demonstrates that a distended bladder can cause reversible pelvic venous congestion and lead to epidural venous plexus engorgement. This phenomenon may mimic disc herniation and/or pathologic venous enlargement as in d-AVF, and familiarity with this clinical entity is crucial to avoid misdiagnosis and to prevent potential unnecessary surgical intervention.

## Learning points

1. Radiculopathy from dilated epidural varices may mimic disc herniation, fistula or mass. Oftentimes, the mimicker is discovered during surgery.
2. We report a case of epidural venous plexus engorgement secondary to a distended bladder, with immediate resolution following bladder decompression.
3. Familiarity with this clinical entity is crucial to avoid misdiagnosis and prevent potential unnecessary surgical intervention.

## Consent

The patient is deceased, and informed consent to publish could not be obtained from the patient or the guardian/next of kin. Exhaustive attempts were made to contact the patient and guardian/next of kin to no avail. The information submitted to the journal has been sufficiently anonymized to protect patient identity.
